# Autism and intention attribution test: a non-verbal evaluation with comic strips

**DOI:** 10.1186/s12991-023-00461-2

**Published:** 2023-08-12

**Authors:** Ilenia Le Donne, Margherita Attanasio, Antony Bologna, Roberto Vagnetti, Francesco Masedu, Marco Valenti, Monica Mazza

**Affiliations:** 1 Department of Biotechnological and Applied Clinical Sciences, University of L’Aquila, Via Vetoio, Località Coppito, 67100 L’Aquila, Italy; 2 Regional Reference Centre for Autism of the Abruzzo Region, Local Health Unit ASL 1, 67100 L’Aquila, Italy

**Keywords:** Autism spectrum disorders, Intention attribution, Psychometric proprieties, Theory of Mind, Clinical utility

## Abstract

**Background:**

Despite autism spectrum disorder (ASD) and mentalization being two words often associated in the literature, the assessment of this ability in individuals with ASD in the clinical setting is still limited. Indeed, there are no standardized Theory of Mind (ToM) tests that are adaptable to different cognitive profiles, such as individuals with language poverty, and intellectual or memory impairments. This study proposes a non-verbal test (Intentions Attribution-Comic Strip Test; IA-CST) to evaluate the ability to infer the intentions of others, a basic component of ToM, in the clinical setting.

**Method:**

In Study 1, the test was administered to 261 healthy individuals and we performed structural validation using Exploratory Graph Analysis. In Study 2, the final version of the test was administered to 32 individuals with ASD to assess the known group validity of the measure by comparing their scores with a sample of IQ-matched controls. Moreover, we performed logistic regression and ROC curve to preliminarily assess the diagnostic performance of the IA-CST.

**Results:**

The IA-CST resulted in a 3-dimension measure with good structural stability. Group comparison indicated that the ASD group shows significantly lower performance in intention attribution but not in inferring causal consequences. The test demonstrated known group validity and that, preliminarily, it is suitable for implementation within the clinical practice.

**Conclusions:**

The results support the IA-CST as a valid non-verbal task for evaluating intentions attribution in the clinical setting. Difficulties in ToM are early and relevant in ASD, so assessing these aspects is valuable for structuring individualized and evidence-based interventions.

## Introduction

Theory of Mind (ToM) is the ability to naturally infer the intentions, beliefs, thoughts and feelings of others, which is useful for predicting their behavior [1, 2] and is one of the main components of social cognition, i.e., a multidimensional construct that refers to the ability to process the social world [3]. ToM is a skill that develops along a continuum and follows defined stages [4, 5], ranging from basic skills (i.e., joint attention) to more complex forms of mentalization (i.e., attributing different mental states to several people). The ability to understand the behavior of others requires an awareness that there is an intention behind an action, a capacity that seems to develop right back in early childhood [6]. Understanding an action first requires identifying what has been done; actions can be identified at a lower level, through details indicating how the action was performed, and at a higher level, through details indicating why and with what effect the action was performed and the effects it had [7]. Recognizing one's actions at the highest level is usually an indication of being aware of one's mind as the cause of the behavior [7, 8]. Action identification makes it possible to keep track of the inference of mental states and this principle can be applied to one's own mental state as well as that of others [7].

Literature suggests that a deficit in planning an action in a specific situation explains the difficulty of mentally representing the intention of that action [9, 10]. This model of functioning has been studied mainly in schizophrenia [11–14]. The ability to understand the intentions of others has also been shown to be impaired in individuals with autism spectrum disorder (ASD) [15–17]. Several studies have suggested that people with ASD have a different way of action processing [16, 18, 19] and would have difficulty in anticipating the actions of others and representing goal-orientated behaviors [19, 20]. This difficulty was evident in the task characterized by illustrated story, known as the “comic strip task”, which required the sequencing of a goal-directed action [19]. The inability to identify and predict actions could be one of the causes of the difficulties in social interaction and the perception of social information in individuals with ASD [21, 22].

Despite the strong association between ASD and deficit in ToM abilities, the literature reports conflicting results [23–25]. Some studies suggest that adults with ASD have significant difficulty in inferring the mental and emotional states of others [26–29]. On the other hand, other studies report similar performances in both individuals with ASD and healthy controls in ToM's tasks [30, 31]. Furthermore, there is a scarcity of data available to demonstrate, at group level, how individuals with ASD, especially adolescents and adults, compare with IQ-matched healthy controls [24]. Several instruments have been developed to evaluate mentalizing ability, such as the Reading the Mind in the Eyes test [26, 32], the Theory of Mind Assessment Scale [33], and the Edinburgh Social Cognition Test [23], whose psychometric properties have been evaluated. However, the use of ToM measures remains mainly confined to the field of research, and their application in clinical practice is still a challenge. In addition, most measures for assessing ToM, such as the Strange Stories [27], require well-developed expressive and receptive language (including, for example, long verbal descriptions and instructions), or involve a huge memory load making it difficult to administer to individuals with ASD that have impaired verbal and cognitive abilities [29, 34, 35].

Several strategies have been suggested to simplify the ToM tasks and facilitate comprehension of the instructions: in some cases, situations similar to the subjects' daily lives were presented; in others, repetition of the story was made available when the patient seemed not to understand; and in yet others, visual aids such as drawings or vignettes were provided [36–39]. The use of vignettes seems to have proved useful in the assessment of ToM in clinical populations, particularly in schizophrenia [40].

We could not find studies in which there is a corresponding task of understanding the intentions used in young adults and adults with ASD. In two of our previous studies [5, 34], we used a comic strip task in children with ASD. In these studies, children were presented with three pictures that told a social story; they were then given two images representing alternative endings and asked to choose the appropriate one [41, 42]. We wondered whether the construction of a similar task using comic strips in which the linguistic and memory component was minimized might be useful in demonstrating that a deficit in intention attribution does not depend on other cognitive deficits. In accordance with Baron-Cohen and collaborators [43], we compared the abilities of the ToM with the understanding of physical causality. Based on these premises, we propose the Intentions Attribution-Comic Strip Test (IA-CST), a test aimed at evaluating the ability to infer characters’ intentions and understand their behaviors. This test can be interpreted as a measure of basic ToM ability and as a precursor of higher-order mentalization skills. Thus, the aim of our study is twofold: (1) to validate a new non-verbal test for the evaluating the attribution of intentions on a large sample, including adolescents and adults (Study 1); (2) to compare the performance of individuals with ASD with IQ-matched controls (Study 2). In a broader perspective, our study aims to provide a standardized tool for the evaluation of a ToM ability that is practical and language-free, to be integrated within the clinical setting to support diagnostic evaluation and intervention planning in ASD.

## Study 1: construction and validation of the IA-CST

### Methods

#### Procedure

Scale validation was performed following three phases [44]. In the first phase, items were constructed and reviewed by experts (2 psychometricians, 1 neuropsychologist, and 1 biostatistician) with extensive knowledge and experience in the field of autism and social cognition. Successively, a convenience sample was used to remove possible confounding items (i.e., items that might have been unclear or difficult to interpret). In the second phase, we performed a structural validation of the scale performing an Exploratory Graph Analysis and a Confirmatory Factor Analysis. Moreover, the relation between performance on the IA-CST and the Advanced Theory of Mind [45, 46], a verbal test of cognitive ToM (concurrent validity), was analyzed.

Lastly, in the third phase reported in Study 2, we preliminarily assess external validation of the new measure assessing known group validity.

The Ethics Committee approved the protocol prior to the recruitment of participants, according to the principles established by the Declaration of Helsinki. Written informed consent and socio-demographic information were provided by all participants, as well as their parents when underage, prior to test administration. Each participant was assessed individually in a quiet room without any distractions. Their responses were registered using paper and pencil. A psychologist was present in the room during the administration to provide any further information if necessary.

#### Measures

##### Raven’s Standard Progressive Matrices

Raven’s Standard Progressive Matrices (SPM) [47] were used to assess non-verbal intelligence and IQ level was calculated following conversion tables. The SPM consists of 60 items, each of which requires the completion of a set of figures with the missing one, from a presented pattern. Each item becomes progressively more difficult, requiring analysis, coding and interpretation skills. We chose to use SPM as its administration is shorter, less demanding and less stressful than typical IQ tests (e.g., Wechsler scales).

##### Advanced Theory of Mind (A-ToM)

A-ToM [45, 46] is an Italian adaptation of a cognitive ToM task (i.e., Strange Stories) that Blair and Cipollotti [45] used and was first proposed by Happé [27]. Happé [27] defined the Strange Stories task as an “advanced” ToM task and proposed that it would be useful for individuals with high-functioning forms of ASD who might otherwise succeed at (first-order) ToM tests. This task includes a two-level investigation of the story protagonist’s mental states, because the stories contain an understanding question and a key question to explain the cause of his/her behavior [48]. In our study, we used the Italian adaptation [46], which consists of a shorter version of 13 stories that describe real events; for correct interpretation, the task requires the subject to go beyond the literal meaning of the text and to draw an inference about the story protagonist’s mental state. A score of 1 is assigned for each item if the comprehension and the justification questions are answered correctly, and 0 otherwise. For more details, please refer to Happé [27] and Mazza et al. [29].

##### Intention attribution-comic strip test (IA-CST)

IA-CST is a comic strip test that evaluates the ability to infer characters’ intentions and understand their behaviors. It consists of six cartoon-like vignettes illustrating a sequence of purposeful actions performed by a character in a daily life scenario. The test also includes a series of items assessing physical causality. For each item, participants are shown three vignettes describing the action or causal relation, after which they are presented with three vignettes each containing an alternative conclusion to the scenario. The participant is asked to choose the correct ending from those proposed. Specifically, the three possible endings represent: (a) the correct ending, which is understandable if the participant can understand the protagonist's intention (or causal relation); (b) a wrong ending very similar to the last picture of the sequence; c) a wrong ending describing an everyday action not associated with the sequence. Correct conclusions indicated by participants represent a score of 1 point, and 0 otherwise.

#### Construction of IA-CST

A total of 38 stimuli were originally constructed by one member of the research team. Stimuli were designed to elicit the participant’s ability to deduce the character’s intention or physical causal inference. Specifically, intention stimuli were designed to try to elicit first- or second-order intentions. After constructing the items, the research team met in order to review each of the proposed stimuli following a qualitative content validity approach. During this meeting, items considered unclear or possible confounders or not an adequate measure of target dimensions were reviewed or discarded. A total of 12 stimuli were removed from the pool, while the remaining were considered adequate by the whole research team. Then, the 26 stimuli were administered to the healthy sample (see Participant section). The percentage of correct responses for each item was calculated. This preliminary analysis led to the discarding of three other items because they presented an extremely high percentage of errors (> 70%); the subsequent review of these items indicated that their correct answers could be difficult to interpret and they were discarded. At the end of this phase, the number of items considered adequate for the subsequent step was 23, henceforth coded from I1 to I23.

#### Participants

A total of 261 healthy individuals (age range in years 14–48, mean chronological age 20.09 ± 4.71, 122 males and 139 females, IQ mean 94.57 ± 4.22), participated in the study during the second phase of the study. They were Italian native speakers and recruited by opportunity from local structures and organizations. If a participant reported a current or past history of substance abuse, neurological and/or psychiatric disorders were excluded from the study. Details of the participants' characteristics are given in Table 1.

**Table 1:** Socio-demographic data and social cognition measures results of the healthy sample

	Group age		Full sample	Statistics	*p*
	14–18	19–48			
*N*	82	179	261		
Gender					
Male (N)	52	70	122	${x}^{2}\left(2\right)=13.35$	**< 0.01**
Female (N)	30	109	139		
Mean age (SD)	15.18 (1.08)	22.34 (3.97)	20.09 (4.71)		
Mean years of education (SD)	10.16 (1.02)	14.97 (1.14)	13.46 (2.49)		
Mean QI (SD)	94.44 (4.46)	94.64 (4.11)	94.57 (4.22)	*F*_1,259_ = 0.123	0.73
Social cognition measures					
A-ToM	9.30 (1.49)	11.26 (1.92)	10.65 (2.01)	*F*_1,259_ = 62.93	**< 0.001**
Total IA-CST	8.45 (0.71)	8.33 (0.68)	8.36 (0.69)	*F*_1,259_ = 1.85	0.17

Significant comparisons (*p* < 0.05) are reported in bold

#### Statistical analysis

Exploratory Graph Analysis (EGA) was performed to investigate items latent factors of the scale. This method was originally proposed by Golino and Epskamp [49]. Within this framework items of the scale are considered nodes of the network and edges between nodes partial correlation coefficients [50]. In this approach, latent constructs are characterized in terms of subnetworks within a wider network described by nodes (item) and edges (partial correlations). The main goal of EGA is to detect clusters of highly connected nodes, according to a separation function, known as modularity [51], and a corresponding optimal separation configuration. This goal is achieved by performing separated random walk explorations which should stochastically converge to the optimal cluster separation if the pattern exists. This approach should enable us to set out the underlying subnetwork structure. Some points of strength of this approach are that its results are comparable, or even better, to other traditional techniques used to detect latent dimensions [49, 50, 52], it can overcome problems related to the choice of a rotation method [53, 54], and it reduces the risk of researcher-related error or bias [50, 53].

A Gaussian Graphical Model (GGM) [55], was estimated through a variant of the least absolute shrinkage and selection operator (LASSO) [56], namely graphical LASSO [57]. This is a regularization technique used to estimate the model and parameters of the GGM [58].

A tuning parameter is set by minimizing an Extended Bayesian Criteria (EBIC) [59], which is an in-index to estimate optimal model fitting [60], which, in turn, is regulated by a parameter γ. EGA implements and algorithm which sets γ based on network resulting connections. For a detailed description please refer to Golino et al. [50].

LASSO reduces at zero edges with little values [60, 61] allowing to limit false-positive edges and returning conservative and replicable results [62]. Number of dimensions were detected using the Walktrap algorithm [63] as proposed by Golino and Epskamp [49]. Then stability of dimensions and items were evaluated through a bootstrap approach to assess the generalizability of results as proposed by Christensen and Golino [64]. A non-parametric bootstrap was performed with 1000 iterations. Descriptive statistics such as median, CI 95% and frequency of numbers of factors were obtained through all the bootstraps. Moreover, structural consistency, i.e., how often the empirical EGA dimension is exactly replicated, and item stability, i.e., each times each item is placed in each dimension, as indicated by Christensen and Golino [64], item stability values ≥ 0.70 were considered as acceptable. Unstable items were then removed, and another model was constructed without those items.

The final model was then further evaluated by performing confirmatory factor analysis (CFA) and goodness of fit was assessed by calculating comparative fit index (CFI), root mean square of error approximation (RMSEA), goodness of fit index (GFI) and Chi-square/degrees of freedom (Chisq/df) where a good fitting was considered by CFI > 0.90; RMSEA < 0.08, GFI > 0.90 and Chisq/df < 3. The internal consistency and reliability were assessed using Cronbach’s α. In addition, we performed Pearson correlation to investigate the relationship between the IA-CST and a verbal test of social cognition (A-ToM).

## Results

**Fig. 1:**
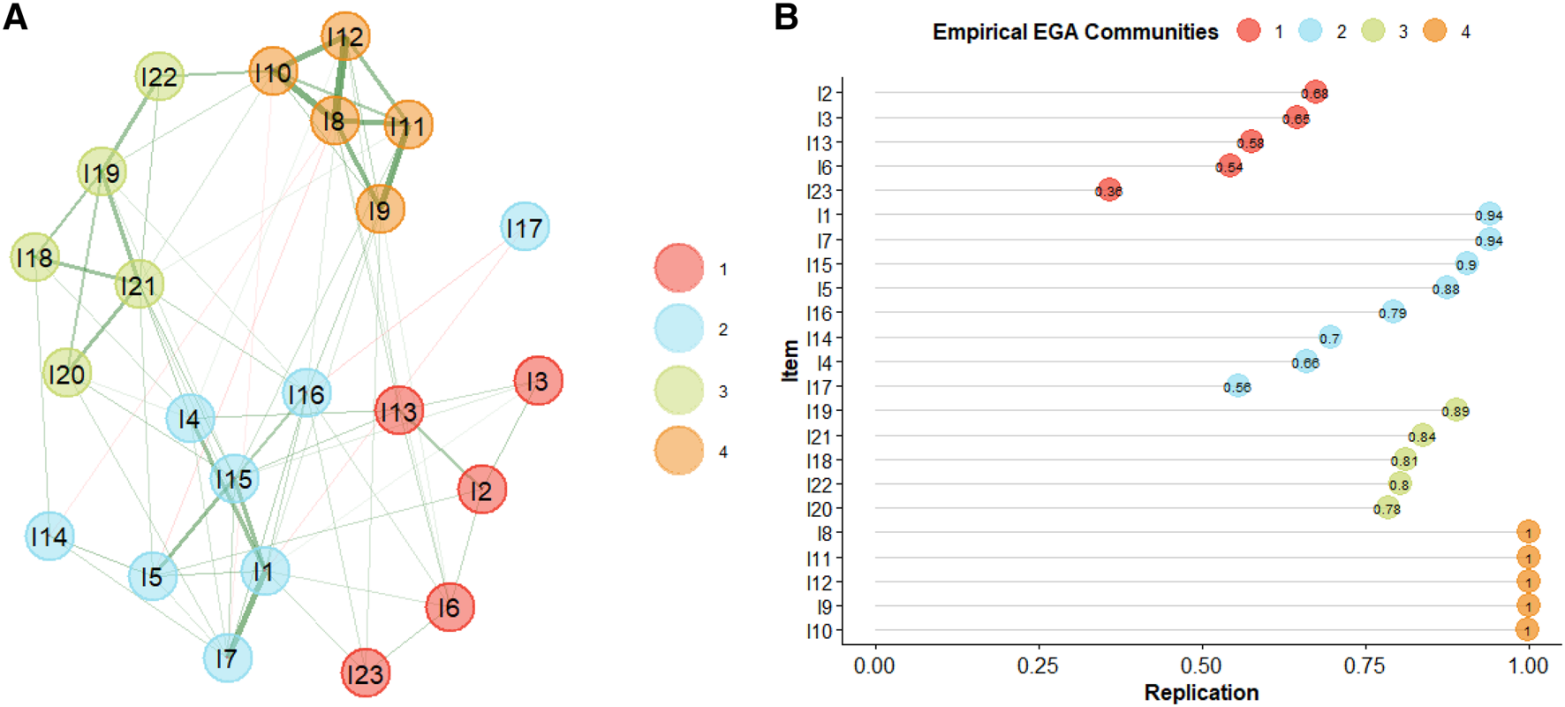
Exploratory graph analysis and item stability results from the first item selection

The first network resulting from EGA is reported in Fig. 1A, the model indicates a 4-dimension solution. The median number of dimensions showed by the bootstrapped networks was 4 (CI 95% [2.54, 5.45]). This analysis indicated that 48% of the networks were characterized by 4 dimensions. In terms of structural consistency, namely how frequent is the occurrence of a dimension, we got the following distribution: dimension 1 was observed 19% times, dimension 2 was 22%, dimension 3 was 60% and dimension 4 was 99%, indicating low structural consistency for all dimensions, except for dimension number 4.

Taken together, these results were interpreted as indicating low structural stability of the networks.

This conclusion was further confirmed by item stability analysis (Fig. 1B) where all of Dimension 1’s items were not stable (< 0.70). These results lead to remove all the items of Dimension 1. Moreover, Dimension 2 showed two unstable items (item stability < 0.70) and one item with stability near the cut-off (I14, item stability = 0.71), we choose to comprehend this item within unstable items, accordingly these three items were removed. Items were removed to find a better structural solution for the network. Then, items from the three Dimensions (excluding Dimension 1) were reviewed to assess the construct represented by each of them. The revision indicated that items of Dimension 2 were evaluating First Order Intention Attribution except for one item which was considered a causal effect item by the research team, thus it was removed from further analysis. Revision of Dimension 3 items indicated that they measure Causal Inference while revision of items of Dimension 4 indicated that they measure Second Order Intention Attribution. Thus, the remaining items were used to conduct a second EGA.

**Fig. 2:**
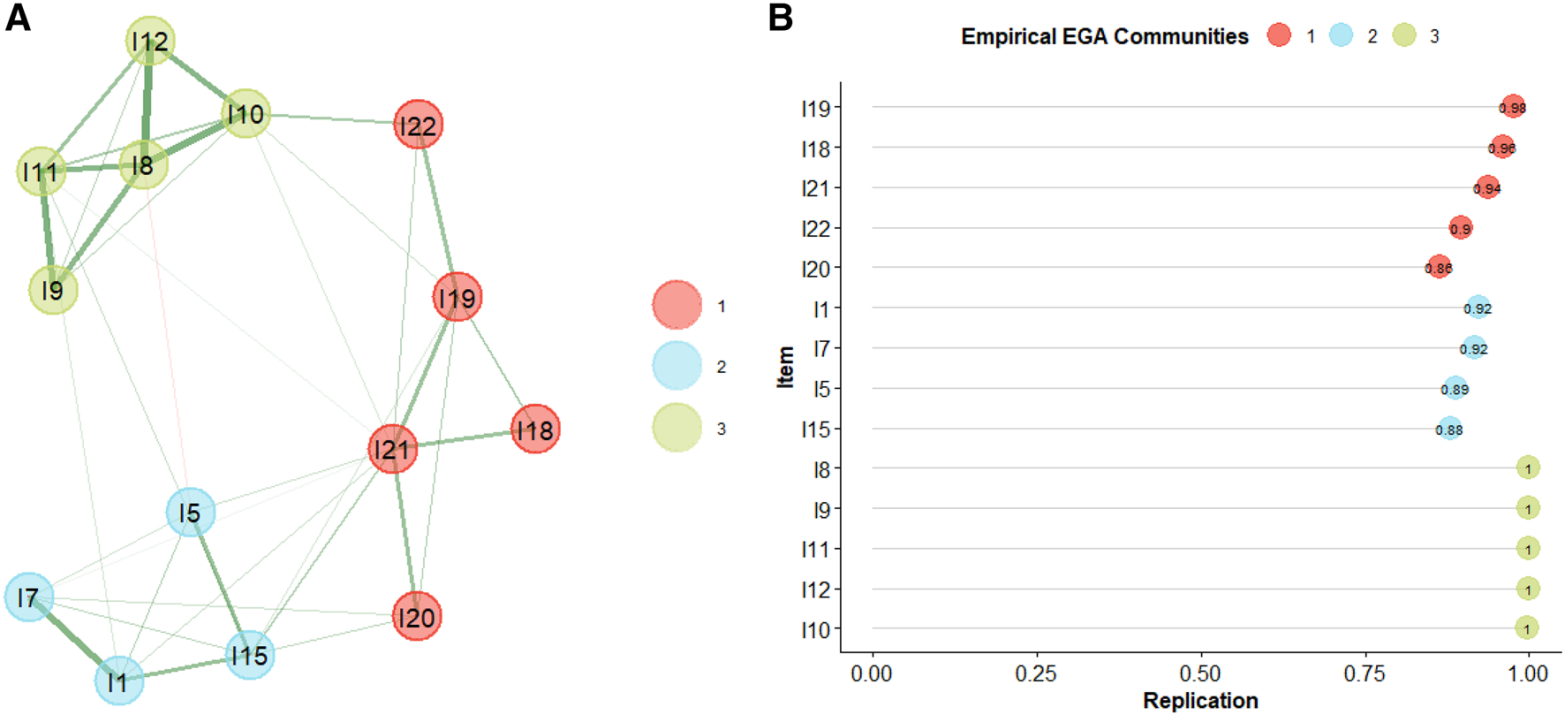
Exploratory graph analysis and item stability results after unstable items removal

The results of the second EGA are reported in Fig. 2A. The model indicates a 3-dimension solution, where Dimension 1 represents Causal Inference, Dimension 2 represents Second Order Intention Attribution and Dimension 3 represents First Order Intention Attribution, according to the previous review. A description of each dimension is reported in Table 2.

**Table 2:** Dimensions’ description of the final network model

Dimension	Description	Vignette example	Item
Causal Inference (Control Condition)	A correct answer implies an understanding of the cause–effect relationship of the objects or people in the scene. Inference of intention is not required to pass these items	The cartoon shows an object X going against an object Y. The correct answer shows Y being moved by object X	I18, I19, I20, I21, I22
First Order Intention Attribution	A correct answer implies an understanding of the character’s intentions. The correct answer, to be indicated by the participant, shows the very moment of the achievement of the goal by the protagonist of the scene	The cartoon shows a person who wants to reach an object beyond his reach. Correct answer depicts the man with the object in his hand	I8, I9, I10, I11, I12
Second Order Intention Attribution	A correct answer implies an understanding of the character’s intentions. The correct answer, to be indicated by the participant, shows an action necessary to achieve the goal, which is not yet reached by the protagonist	The cartoon shows a person who wants to reach an object beyond his reach. Correct answer depicts the man while performing an action that will enable him to achieve the objective	I1, I5, I7, I15

Bootstrap results indicated a median of 3 dimensions with a relative narrow CI 95% [2.34, 3.65], this number of dimensions was obtained in 88% of the simulated networks. Structural consistency results indicated good stability for Dimension 1 (78%), Dimension 2 (85%), and Dimension 3 (99%). Taken together these results were interpreted as indicating good structural stability of the networks. This conclusion was also confirmed by item stability results where all items showed high item stability values (all > 0.80, please refer to Fig. 2B). Thus, a 3-dimension structure for the items considered in the analysis could be considered as a stable and replicable solution.

Finally, as further confirmation, the three-dimension model represented in Fig. 2, was further inspected through CFA. The CFA model fitting of data resulted in CFI = 0.86; RMSEA = 0.07, GFI = 0.91 and Chisq/df = 2.55. Considering that RMSEA, GFI and Chisq/df indicated a good fitting, while CFI reached a close value to 0.90, we interpreted this result as an acceptable fitting of the data. Thus, based on EGA results and CFA results we established as final structure of the IA-CST the three-dimension model, shown in Fig. 2.

In summary, the network analysis performed two structural stability analyses, one referred to the number of dimensions representing our data, and another one to the item stability, letting us understand how much chance influenced the latent items structures.

### Final scale: the IA-CST

The final scale consists of 14 stimuli divided into three subscales: Causal Inference (C IA-CST), First Order Intention (1st IA-CST) and Second Order Intention (2nd IA-CST). The total scores for each subscale that can be obtained range from 0 to 5 for C IA-CST, 0–5 for 1st IA-CST and 0–4 for 2nd IA-CST. For the Causal Inference, we calculated the 5th percentile as a threshold; the results indicated that to proceed to the other two conditions, the subject should correctly answer at least 4 out of 5 control items.

Moreover, a total score of Intention Attribution abilities was obtained summing the dimensions of First and Second order Intention attribution scores (Total IA-CST = 1st IA-CST + 2nd IA-CST, range 0–9). Reliability for the Total IA-CST was good (α = 0.7) and very good for 1st IA-CST subscale (α = 0.84); an acceptable reliability was found for the 2nd IA-CST (α = 0.6) and C IA-CST (α = 0.6) subscales. Correlation results show that subscales of First and Second order Intentions significantly correlated with A-ToM total score (*r* = 0.324, *p* = 0.017 – *r* = 0.390, *p* = 0.004, respectively). In addition, the total score of Intention Attribution abilities correlated with the total score of the A-ToM (*r* = 0.492, *p* < 0.001).

## Study 2: clinical validity

### Methods

#### Participant

Thirty-two Level 1 ASD (mean chronological age 18.53 ± 2.53, mean IQ 94.07 ± 10.58, mean years of education 10.84 ± 1.61) were recruited by the Reference Regional Centre for Autism (CRRA) [65]. The diagnosis was made according to Autism Diagnostic Observation Schedule-2 [66] and the Diagnostic and Statistical Manual of Mental Disorders-5th [67] by experienced clinicians. ASD participants were excluded from the study if they presented concurrent psychiatric or medical conditions and cognitive impairment. Moreover, 32 typically developing (TD) participants (mean chronological age 19.19 ± 3.01, mean IQ 95.70 ± 4.44, mean years of education 12.63 ± 2.83) were matched with ASD by IQ, age, and gender.

#### Statistical analysis

To preliminary assess the IA-CST potential clinical use and known group validity, the resulting IA-CST task from the previous analysis was then assessed to a group of 32 TD and 32 ASD matched by IQ, gender, and chronological age. IA-CST scores were compared through the Mann–Whitney test, and group differences were assessed with a non-parametric test as, given sample sizes, we performed a Shapiro–Wilk test which indicated that the IA-CST measures for both groups did not follow a normal distribution. We performed Spearman correlations between IQ, age and years of education with IA-CST scores for both groups to explore dimensions associated with the measure. A binomial logistic model was implemented to understand if scores on the IA-CST could be used to predict ASD diagnosis, then diagnostic performance was evaluated by Receiver Operating Characteristic curve (ROC), the optimal cut-off was estimated by Youden Index, AUC accuracy values between 0.90–1.00 were considered as excellent, 0.80–0.90 as good accuracy, 0.70–0.80 as fair accuracy and 0.70–0.60 as poor accuracy [68]. Then an EGA approach was also performed with the ASD sample to assess IA-CST dimensions within the clinical group, however, the best fitting model was an empty network, thus results are not reported.

Analysis was performed using R [69], the EGAnet package version 0.9.9 [70], and the lavaan package, version 0.5–12 BETA [71]. ROC curve analysis was performed with SPSS 25.0 [72].

## Results

Thirty-two young adults with ASD (28 males and 4 females) were compared with 32 TD participants (25 males and 7 females). Groups were matched by IQ (*t*(62) = 0.80; *p* = 0.42) and did not show differences regarding chronological age (*t*(62) = 0.94, *p* = 0.34) and gender (*χ*^2^ (1, *N* = 64) = 0.98, *p* = 0.32). The two groups show a difference in years of education (*t*(62) = 3.09, *p* < 0.01). Demographical and clinical information are reported in Table 3. Then, according to item clusters reported in Fig. 2 and in Table 1, groups were then compared on the total score of Causal Inference (C IA-CST), First Order Intention (1st IA-CST), and Second Order Intention (2nd IA-CST) of the IA-CST.

**Table 3:** Demographic data of the samples and clinical information regarding ASD

	ASD (*N* = 32)	TD (*N* = 32)	Test statistic	*p*
Gender (M; F)	28; 4	25; 7	χ^2^ (1, *N* = 64) = 0.98	0.32
Mean chronological age (SD)	18.53 (2.53)	19.19 (3.01)	*t*(62) = 0.94	0.34
Mean IQ (SD)	94.07 (10.58)	95.70 (4.44)	*t*(62) = 0.80	0.42
Mean years of education (SD)	10.84 (1.61)	12.63 (2.83)	*t*(62) = 3.09	** < 0.01**
Clinical information				
Mean year of first diagnosis (SD)	10.68 (5.34)	–	–	–
ADOS-2 (Module 4)		–	–	–
Communication	4.33 (1.63)	–	–	–
Social interaction	8.66 (3.35)	–	–	–
Communication + social interaction	13.00 (4.62)	–	–	–
Stereotyped behaviors and restricted interests	0.40 (0.51)	–	–	–

Significant comparisons (*p* < 0.05) are reported in bold

IA-CST scores for both groups are presented in Table 4. Results indicated a significant difference in 1st IA-CST (*U* = 318.00, *z* = − 2.95, *p* = 0.003) where the ASD group showed lower scores (Mdn = 4.50) compared to the TD group (Mdn = 5.00) and, a significant difference in 2nd IA-CST (*U* = 292, *z* = − 3.40, *p* < 0.001) where the ASD group showed lower scores (Mdn = 3.50) compared to the TD group (Mdn = 4.00); a significant difference in Total IA-CST (*U* = 251.50, *z* = − 3.66, *p* < 0.001) where the ASD group showed lower scores (Mdn = 8.00) compared to the TD group (Mdn = 9.00). Results did not indicate a difference regarding C IA-CST (*U* = 386.00. *z* = − 1.82, *p* = 0.068) within the comparison between ASD (Mdn = 5.00) and TD (Mdn = 5.00).

**Table 4:** IA-CST scores comparison between ASD and TD groups, and descriptive statistics according to gender

	ASD group		TD group		*Z*	*p*
	Total sample (*N* = 32)		Total sample (*N* = 32)			
	Mean (SD)	Median (1st–3rd quartile)	Mean (SD)	Median (1st–3rd quartile)		
C IA-CST	4.54 (0.76)	5 (4–5)	4.83 (0.45)	5 (5–5)	− 1.82	0.068
1st IA-CST	4.28 (0.92)	4.50 (4–5)	4.83 (0.37)	5 (5–5)	− 2.95	**0.003**
2nd IA-CST	2.96 (1.25)	3.50 (2–4)	3.87 (0.34)	4 (4–4)	− 3.40	**< 0.001**
Total IA-CST	7.25 (1.84)	8 (6–9)	8.70 (0.46)	9 (8–9)	− 3.66	**< 0.001**

IA-CST score by gender						
	ASD males (*N* = 28)		ASD females (*N* = 4)			
	Mean (SD)	Median (1st–3rd quartile)	Mean (SD)	Median (1st–3rd quartile)		
C IA-CST	4.48 (0.80)	5 (4–5)	5.00 (0.00)	5 (5–5)		
1st IA-CST	4.25 (0.96)	4.5 (4–5)	4.50 (0.57)	4.5 (4–5)		
2nd IA-CST	2.96 (1.26)	3.5 (2–4)	3.00 (1.41)	3.5 (1.5–4)		
Total IA-CST	7.21 (1.87)	8 (6–9)	7.50 (1.91)	8 (5.5–9)		

	TD males (*N* = 25)		TD females (*N* = 7)			
	Mean (SD)	Median (1st–3rd quartile)	Mean (SD)	Median (1st–3rd quartile)		
C IA-CST	4.86 (0.35)	5 (5–5)	4.81 (0.54)	5 (5–5)		
1st IA-CST	4.86 (0.35)	5 (5–5)	4.81 (0.40)	5 (5–5)		
2nd IA-CST	3.86 (0.35)	4 (4–4)	3.87 (0.34)	4 (4–4)		
Total IA-CST	8.73 (0.45)	9 (8–9)	8.68 (0.47)	9 (8–9)		

*Z* statistics are obtained from Mann–Whitney test; significant differences (*p* < 0.05) are reported in bold

Spearman correlations are reported in Table 5, results indicated that only IQ for the ASD group was significantly correlated with C IA-CST (*r* = 0.42; *p* = 0.02).

**Table 5:** Spearman correlations for both groups between IA and age with IA-CST scores

	C IA-CST	1st IA-CST	2nd IA-CST	Total IA-CST
TD group				
Chronological age	0.20	− 0.01	0.26	0.17
IQ	0.02	− 0.10	− 0.05	− 0.12
Years of education	0.13	− 0.02	0.25	0.17
ASD group				
Chronological age	− 0.06	0.03	0.12	0.05
IQ	**0.42**	0.23	0.23	0.32
Years of education	0.14	0.17	0.13	0.14

Significant correlations (*p* < 0.05) are reported in bold

Finally, logistic regression was carried out to evaluate the effect of Total IA-CST on the likelihood on receive an ASD diagnosis or not. The model was statistically significant if compared to a null model (*χ*^2^(1) = 19.60, *p* < 0.001), furthermore, it explained 36% of the variation in group membership (according to Nagelkerke *R*^2^) and it correctly predicted 68% of the sample. Total IA-CST was a significant predictor (*β* = − 1.22, SE = 0.40, Wald’s *χ*^2^(1) = 9.24, *p* = 0.002) indicating that this score could help in differentiating between ASD and TD groups. Based on this outcome, we constructed a ROC curve with Total IA-CST score to address its diagnostic performance. ROC curve of Total IA-CST results indicated a fair classification accuracy (AUC = 0.75, SE = 0.06, *p* = 0.001, CI 95% [0.63, 0.87]), where the best cut-off for an ASD diagnosis was a score < 9 (sensitivity = 71%, specificity = 66%, overall correct classification = 68%).

## Discussion

Theory of Mind is widely studied in ASD research, and several instruments have been developed to evaluate this ability [29, 34, 73]. Moreover, most standardized tests of ToM require well-developed expressive and receptive language skills and cannot be used among individuals with little or no verbal ability [29, 34, 35]. This leads to the exclusion of a sub-group of individuals with ASD (those with moderate-or-severe language impairments and/or individuals with intellectual disabilities) for the assessment of ToM abilities whose deficit is considered one of the main characteristics of this clinical condition [3, 26, 35, 74]. Furthermore, many ToM tasks are long or complex, so they could be difficult to apply in clinical settings [75]. The present study was conducted to contribute to evaluating the reliability and validity of a non-verbal test, the IA-CST, that could be introduced in clinical practice to support diagnostic evaluation. The IA-CST consists of a series of cartoon-type stimuli to assess intentionality and causal inference (control condition). To the best of our knowledge, currently, there are no standardized batteries or single tests that allow an assessment of non-verbal attribution of intentions.

Results from structural analysis of the IA-CST indicated unstable items, which were removed, and a three-dimension model structure as optimal; CFA further confirmed this solution, indicating that it was suitable for our sample. Thus, we obtained three subscores, namely First Order Intention Attribution, Second Order Intention Attribution and Causal Inference, as the control score, then a Total Score of Intention Attribution could be calculated by summing the two intention attribution scores. Specifically, for items investigating First Order Intention Attribution, the story is constructed so that the correct ending shows the moment when the character achieves the goal (e.g., the character has the object he wanted in his hand); in Second Order Intention Attribution, the correct ending shows the character performing the action necessary to achieve the goal. The Causal Inference condition does not involve an inference of intentions but an understanding of the cause–effect relationship of objects or people in the scene (e.g., an object as it falls). We suggest using the score obtained in the Causal Inference condition as a control score, which allows access to the intention attribution series if the subject is able to identify at least 4 of the 5 items. Furthermore, our results show that the IA-CST shows good concurrent validity. Although we found significant but weak correlations between the two subscales of First and Second Order Intentions with the A-ToM, the Total Score of the Intentions Attribution showed a moderate correlation with the A-ToM. These results are consistent with the hypothesis that the ability to understand the intentions of others by observing their actions is a prerequisite for the ability to produce reasoning about the mental state, and explain the thoughts and feelings of others, as measured by the A-ToM [1, 76]. In fact, according to Happé and Frith [4], the construct of social cognition can be understood as a complex network that includes distinct components, such as agent identification, self-processing and mental state attribution. All these components are interconnected and influence the development of appropriate social behaviors [4, 77–80]. Our results demonstrate how the different components of social cognition, as measured by the tests used, represent different but related skills and are involved in understanding social agents and social interactions. In particular, we supported the hypothesis that one component, such as action recognition and intention attribution assessed by the IA-CST, may be a (sub)component of another, such as mental state attribution and empathy [79]. It is known that in ASD individuals there is an atypical development of the different (sub)components of social cognition [80], so having specific measures assessing single abilities represents an added value in both the research and clinical field. In Study 2, we preliminarily assessed the clinical validity of the IA-CST and compared the performance of the clinical group, ASD individuals, with IQ-matched controls. The group comparison indicated that ASD individuals show no difficulty in the control condition compared to the IQ-matched group. In contrast, the ASD group shows a significantly lower performance in the two series investigating intention attribution. This confirms that the IA-CST is a useful test with which to identify subtle impairments in intention attribution, which is independent of the comprehension of physical events but is related only to mentalizing abilities. In fact, our results showed that individuals with ASD are significantly impaired in the intention attribution conditions, highlighting an impairment in the mechanisms necessary to understand the intentions of others. Since individuals with ASD are known to show difficulties in social cognition processes [27, 43, 77, 79], one would expect that even in the IA-CST, individuals with ASD would experience difficulties, so this finding indicates the known group validity of the task. Regression analysis confirms this evidence, demonstrating that the ability to attribute intentions is a significant predictor in differentiating between ASD and TD individuals. Further confirmation is provided by ROC analysis, which supports the effectiveness of the IA-CST in discriminating, with a fair level of accuracy, between the ASD and the TD group. The best sensitivity and specificity of the IA-CST, and therefore a higher probability of identifying individuals with ASD, were obtained with a score < 9 (best cut-off). Taken together, these results suggest the potential for using the test in clinical practice. The IA-CST would provide an additional tool during the diagnostic process, incorporating, and placed alongside measures considered to be the gold-standard for the diagnosis and assessment of autism. Standard neuropsychological assessments and diagnostic procedures often lack information on social cognition abilities and, as a result, do not provide appropriate indications for the treatment of these deficits [81]. Impairments in ToM abilities are early and relevant in ASD, so assessing these aspects, along with symptomatic, cognitive, functional, and adaptive features, is also valuable for structuring interventions.

We propose an instrument that is easy to administer and allows for the assessment of a basic ToM ability necessary for the development of higher-level skills. The assessment of the different components of social cognition in autism is important for the delineation of a functioning profile of the individual, highlighting strengths and weaknesses. Currently, the interventions for ASD adolescents and adults most frequently reported in the literature [82] are mainly aimed at improving communicative-relational abilities. However, whether the treatment does not consider the potential impairment of more basic abilities, the risk is that interventions on higher-level abilities will not be effective. The construction of interventions must therefore be individualized, evidence-based, and targeted to the specific impaired skill. The use of specific tests, such as the one we have proposed, also provides valuable information for the structuring and follow-up of interventions for low and medium functioning individuals.

We are aware that our study has some limitations. One of these is the relatively small ASD sample sizes; future studies should explore differences and the diagnostic performance of the IA-CST with larger sample sizes. Moreover, our preliminary results indicated a fair level of accuracy in terms of classification, suggesting its potential for use in clinical-diagnostic assessments. There are gold-standard instruments that clearly the IA-CST cannot replace, but it can be used as an integrative tool to assess another dimension of individual functioning. From this perspective, we would like to underline that a strength of the IA-CST is its short length (14 items in total) and the possibility of administering it to people with poor verbal skills. Our test involves visual-perceptual processing skills. The literature often reports that individuals with ASD have high levels of visual discrimination and perceptual functioning [83, 84], however, this aspect remains controversial [85, 86]. In fact, some studies have shown that individuals with ASD have a deficit in visual processing [85–87]. For example, it has been suggested that atypical gaze patterns in ASD individuals may affect the ability to understand the observed actions, primarily due to abnormalities in visual attention [86, 88]. The population with ASD is largely heterogeneous, so it is likely that, for some individuals with ASD, the use of pictures may facilitate performance, while for others their use may be a disadvantage [86]; when interpreting results, it is important to bear this in mind. One of the fundamental tasks of the clinician is to choose the most appropriate tests based on the characteristics of the individual concerned. During the evaluation process, the clinician should consider any additional relevant investigations in order to fully understand and properly interpret the results.

Among the limitations, we should report that the validation process could be further expanded. The significant correlations that emerged between the IA-CST and the A-ToM were weak/moderate, so this should be further investigated. Moreover, in our framework, items were reviewed by experts; then, through EGA we assessed the internal structure, and we evaluated its ability to discriminate a sample that is known to be impaired in the construct that the instrument is intended to measure.

Furthermore, although the absolute (RMSEA, GFI) and parsimonious (Chisq/df) fit indices reach the commonly accepted criteria, the incremental fit index (CFI) is just below the proposed cut-off (> 90) [89] and, from a broader perspective, we aim to improve it, although these indices should not be interpreted as binary judgements, but rather as an approximation of the data to the model that is more realistic than a perfect fit [90]. Even if our results provide evidence in favor of the validity of the proposed measure, further studies should evaluate additional aspects, for instance, assessing test–retest reliability, predictive validity, and other types of external validity. Indeed, the process of validating a measure consists in obtaining a lot of evidence in favor of its validity. Future studies should overcome these limitations and extend the application of the test also to individuals with ASD at lower functioning.

## Data Availability

The datasets used and analyzed during the current study are available from the corresponding author on reasonable request.
